# The Mental Well-Being of Italian Adolescents in the Last Decade through the Lens of the Dual Factor Model

**DOI:** 10.3390/children9121981

**Published:** 2022-12-16

**Authors:** Michela Bersia, Lorena Charrier, Paola Berchialla, Alina Cosma, Rosanna Irene Comoretto, Paola Dalmasso

**Affiliations:** 1 Department of Public Health and Pediatrics, University of Torino, Via Santena 5 bis, 10126 Torino, Italy; 2 Post Graduate School of Medical Statistics, University of Torino, Via Santena 5 bis, 10126 Torino, Italy; 3 Department of Clinical and Biological Sciences, University of Torino, Regione Gonzole 10, 10043 Orbassano, Italy; 4 Department of Sociology, Trinity College Dublin, 3 College Green, Dublin 2, Ireland; 5 Sts Cyril and Methodius Faculty of Theology, Olomouc University Social Health Institute, Palacký University in Olomouc, Univerzitní 22, 779 00 Olomouc, Czech Republic

**Keywords:** mental well-being, adolescents, mental health, Dual Factor Model, psychological symptoms, life satisfaction, gender, HBSC

## Abstract

(1) Background: In Italy, the components of adolescents’ mental well-being (psychological symptomatology and cognitive perception of life satisfaction) showed different temporal trends, suggesting the adoption of a multidimensional conceptualization. We aimed to assess temporal patterns and provide additional insights into Italian adolescents’ mental well-being in the last decade by adopting the Dual Factor Model; (2) Methods: We used nationally representative samples of Italian students (n = 165,000) aged 11, 13, and 15 years across the three more recent Italian Health Behaviour in School-aged Children (HBSC) surveys. Two measures of mental well-being were used: life satisfaction (LS, indicator of positive subjective well-being) and psychological health complaints (PHC, indicator of mental illness); (3) Results: Our study showed that the overall sample has been moving from a Complete Mental Health (Flourishing) to an Incomplete Mental Illness (Struggling) condition. Among 13- and 15-year-old girls, a jump from one to the other mental condition was observed in the 2014–2018 and 2010–2014 time periods, respectively; (4) Conclusions: Our findings suggest that Italian adolescents, especially older girls, have been shifting from Complete Mental Health to Incomplete Mental Illness in the last decade. Further research is needed to investigate this breaking up of the connection between psychological symptomatology and cognitive perception of life satisfaction.

## 1. Introduction

According to the World Health Organization (WHO), 10–20% of adolescents globally experience mental illness, largely underdiagnosed and undertreated [1]. Such evidence demands surveillance projects exploring the wide concept of mental well-being among general population settings (e.g., schools) [2], such as the Health Behaviour In School-Aged Children (HBSC) survey and the Youth Risk Behavior Surveillance System (YRBSS) [3,4]. In 2018, the HBSC estimated that from 30% to 41% of 11-,13-,15-year-olds adolescents reported multiple psychosomatic health complaints and average life satisfaction from 7.4 to 8.3 out of 10 [5]. There is systematic evidence that high school stress and low social support negatively impact mental well-being, whereas good family support promotes positive mental well-being [6,7].

In recent years, the conceptualization of mental health using the Dual Factor Model is receiving major support and attention [8,9,10,11]. This model considers mental health as a multidimensional construct where the absence of mental health problems represents a necessary but insufficient condition for mental health. As such, positive subjective well-being and mental illness are more than just opposite ends of the same continuum, and research ought to assess both components to capture mental health in a comprehensive manner. Based on this conceptualization, the previous research has derived four distinct mental states [12,13]: Flourishing or Complete Mental Health (low mental illness and high subjective well-being), Languishing or Incomplete Mental Health (low mental illness and low subjective well-being), Struggling or Incomplete Mental Illness (high mental illness and high subjective well-being) and Floundering or Complete Mental Illness (high mental illness and low subjective well-being). Good mental health is achieved when two conditions are satisfied: high subjective well-being and the absence of mental illness. These two factors lie on separate but related dynamic continua [10,11,13]. Previous works have provided some applications of the Dual Factor Model to adolescents (e.g., in Canada and US) showing a high cross-national variability [14,15], so country-specific research is needed. 

Talking about temporal trends, the international picture is controversial, showing a not homogenous pattern [16,17]. However, in some countries (e.g., Sweden, the UK, Italy, Norway, and Canada) an increasing trend of psychological health complaints was already registered, while life satisfaction showed an unclear pattern [18,19,20,21,22].

In Italy, a previous study revealed different temporal trends (from 2010 to 2018) of adolescents’ mental well-being indicators: a mean yearly increase from 2% to 6% in multiple psychological health complaints and overall stability of life satisfaction [22]. These findings suggested the need to provide a more comprehensive key to reading the results. 

This study aimed to provide a wider interpretation of the temporal patterns of mental well-being among Italian adolescents by applying the Dual Factor Model conceptualization.

## 2. Materials and Methods

### 2.1. The HBSC Survey 

Italian data were retrieved from 2010, 2014, and 2018 Health Behaviour in School-aged Children (HBSC) surveys [3,23]. Representative samples of 11-, 13-, and 15-year-old students were recruited from school classes throughout all Italian regions, using standardized and self-administered questionnaires to collect information about a wide range of topics [3,23]. More detailed information can be found in previous work on the same data [22].

### 2.2. Demographic Variables

Age. Students reported the year and month in which they were born. Based on this information, an age variable was computed.

Gender. Students reported whether they are a boy or a girl.

### 2.3. Well-Being Variables

Life satisfaction (LS). A continuous cognitive scale was used to measure well-being: “Here is a picture of a ladder. Suppose the top of the ladder represents the best possible life for you and the bottom of the ladder the worst possible life. Where on the ladder do you feel you stand at the present time?”. The scale ranged from 0 (the worst possible life) to 10 (the best possible life) [24].

Psychological health complaints (PHC). The PHC scale belongs to the HBSC Symptom Checklist (HBSC-SCL). The students were asked how frequently they experienced certain psychological symptoms in the last 6 months (feeling down, being irritable, nervous, and sleeping difficulty). Each item was assessed on a 5-point scale from 1 (i.e., “about every day”) to 5 (i.e., “rarely/never”): as elsewhere [17], a global 0–16 continuous measure was created summing the scores of the scale items (0–4), after having reversed (e.g., 1 = ”rarely/never” and 5 = ”about every day”) and rescaled (from 1–5 to 0–4) the original ones. The adopted 0–16 continuous measure indicates an increasing number and intensity of psychological health complaints.

The psychological subscale showed acceptable test–retest reliability, internal consistency (Cronbach’s α = 0.73), and convergent validity, as it was correlated highly with emotional problems (r = 0.79) and moderately with emotional well-being (r = 0.48) [25]. As is also suggested from some previous evidence [20,26,27], the PHC subscale was adopted separately from the somatic one since the two-factor model fitted the data best according to the Confirmatory Factor Analysis on our data [22]. Moreover, even if it correlated to the psychological factor (0.40–0.83 across countries) [25,26,28], the somatic subscale does not distinguish between purely physical and psychosomatic origins. Therefore, it was decided to adopt the psychological subscale exclusively as the indicator of mental illness to give more priority to specificity than sensibility. In fact, the HBSC-SCL psychological subscale was observed to detect the first signs of further mental conditions in early adulthood, and it is also a valid screening tool for assessing suicidal risk [29,30].

### 2.4. Application of the Dual Factor Model

We used the conceptualization previously utilized in Venning [12], adapted from Keyes [10,13], describing four distinct mental conditions: Flourishing, Struggling, Languishing, and Floundering. In the present study, these conditions were identified using validated HBSC measures of mental well-being [31]. More specifically, two variables were considered as indicators of the two dimensions of the Dual Factor Model: LS was used as a proxy of subjective well-being, while PHC was adopted as a measure of mental illness, similar to some previous literature [14,32].

### 2.5. Statistical Analyses

The Dual Factor Model was applied using the LS cut-off of 6 recommended by the HBSC protocols, while for PHC, no cut-offs on the continuous 0–16 scale were available [5,31]. A sensitivity analysis was performed on the PHC scale using as gold standard the recommended cut-off for the dichotomized factorial variable, meaning to present “≥2 symptoms more than once a week”. A cut-off value of 8 out of 16 was established, using as a criterion the maximization of the ROC (Receiver Operating Characteristic) curve (0.93; 95% CI (Confidence Interval): 0.93;0.93) with a sensitivity of 92.3% (95% CI: 92.1; 92.5) and a specificity of 93.3% (95% CI: 93.2; 93.5). The sensitivity analysis results and the adopted frame of the Dual Factor Model can be seen in Figures S1 and S2 (Supplementary File). Means and SDs were calculated on the LS and PHC variables across waves. Due to interaction, descriptive analyses were stratified by gender and age. All the analyses were performed using statistical software Stata 16.1 (StataCorp College Station, TX, USA: StataCorp LLC), considering survey design effects (including stratification, clustering, and weighting).

## 3. Results

**Figure 1:**
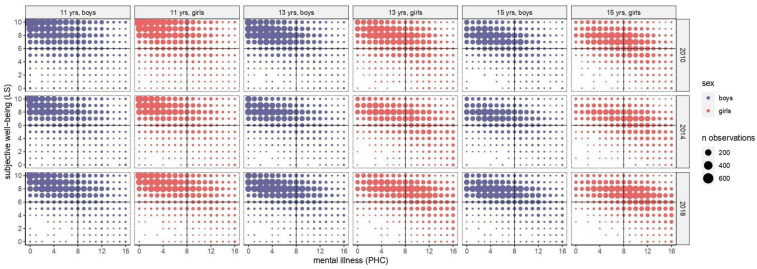
Distribution of observations in the Dual Factor Model frame across time. Stratification by gender and age. Abbreviations. LS: life satisfaction; PHC: psychological health complaints.

More than 165,000 students participated in the survey in three waves (35.6% in 2010, 28.8% in 2014, and 35.6% in 2018) with similar gender and age distribution (35.3% and 28.5% 13- and 15-year-olds, respectively; 49.6% girls). Figure 1 shows the distribution of observations in the Dual Factor Model frame across the three HBSC surveys. Most of our adolescents’ sample was allocated to the two areas with high LS: Flourishing and Struggling.

Furthermore, we identified gender and age-specific patterns: girls and older adolescents were more likely to be in the Struggling area (with high PHC) compared to boys and the youngest age group (i.e., 11-year-olds) who were present mainly in the Flourishing condition (with low PHC). Moreover, these patterns seem to increase over time, as shown in Table 1. Globally, the mean subjective well-being (LS) was steady between 2010 and 2018 inside groups. On the other hand, PHC increased in all age and gender groups from 2010 to 2018: especially among girls, the average PHC escalated from 5.3 to 6.4 at 11 years old, from 6.6 to 8.2 at 13 years old, and from 7.5 to 9.1 at 15 years old.

**Figure 2:**
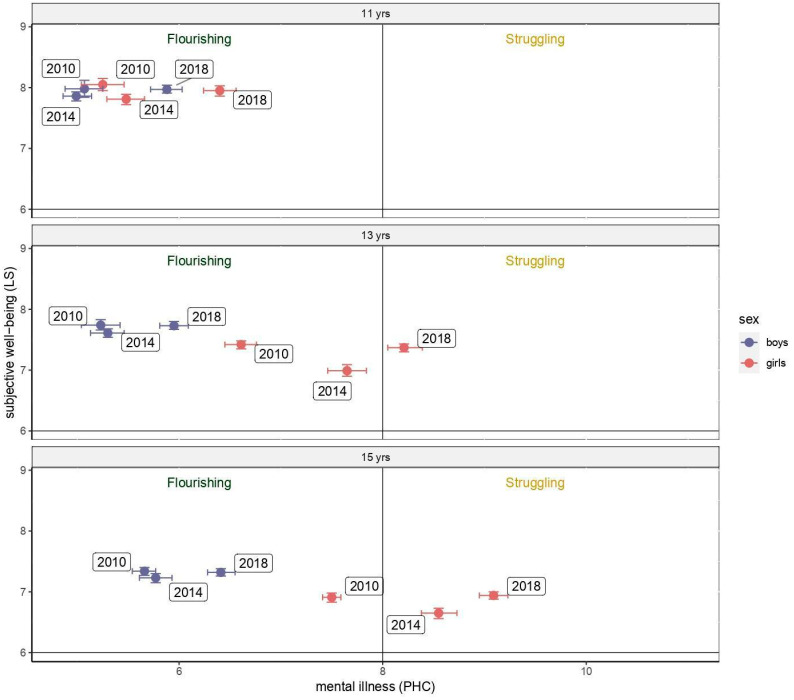
Change over time of the placement of the gender-age groups in the Dual Factor Model frame. PHC and LS as continuous variables. Abbreviations. LS: life satisfaction; PHC: psychological health complaints.

Figure 2 reports the temporal trends of gender and age groups into the Dual Factor Model frame in terms of means and 95% confidence intervals. A shift from the Flourishing towards the Struggling area was observed across time among all gender and age groups. In particular, a skip from one to the other region was seen in the 2010–2014 and 2014–2018 time periods, respectively, among 15- and 13-year-old girls. Such a shift was also seen in the observations’ distribution over time (Figure 1). Furthermore, among 13- and 15-year-old girls, a shift was also reported between the two bottom areas with low LS: from the Languishing (with low PHC) towards the Floundering (with high PHC) condition.

## 4. Discussion

In our previous work [22], we observed an increase in psychological health complaints and steady life satisfaction in the last decade among Italian adolescents. This finding addressed the need to adopt multidimensional conceptualizations of mental well-being, contemplating different but correlated temporal trends of the involved elements [8,9], as observed previously in several other countries [16,22]. According to the Dual Factor Model, the present work found that these different patterns account for a gradual shift from a state of Flourishing, of complete mental health, towards a Struggling condition, of incomplete mental illness. This major concern affects adolescents independently of age and gender. However, 13- and 15-year-old girls seem to be the most affected, showing a skip to the Struggling condition area, respectively, from 2014 to 2018 and from 2010 to 2014.

In Italy, future studies should investigate the implications of this relevant change in adolescents’ mental health. In Canada, the Struggling condition was associated with lower levels of family and school social support, lower academic performance, higher peer support, and increased risk behaviors (i.e., drugs and alcohol consumption) compared to Flourishing one [14]. Peers might have a negative effect in pressuring adolescents in the Struggling state to engage in risk behaviors [14,33]. Adolescents’ previous longitudinal studies report that the Struggling condition could be at higher risk of shifting into the Floundering area (i.e., with low subjective well-being and high mental illness) within 6–12 months compared to the Flourishing state [15,34,35,36,37]. The Floundering condition seems to be associated with low social support (i.e., family, peers, scholar) and school performance and the worst health outcomes in terms of both psychological (i.e., self-worth, external locus of control) and physical health [8]. To our knowledge, no evidence is now available exploring the differences in the social media use style in people belonging to different mental conditions according to the Dual Factor Model, an area that warrants further investigation.

Previous research on gender differences argues that adolescent girls have a higher vulnerability and a sharper upward slope for several mental conditions than boys [16,20,21,38,39,40]. When accounting for the specific Italian context, we could argue that the gender effects observed in this study could be attributed, among others, to the increasing perception of schoolwork pressure and the increase in problematic social media use in the last decade. All these factors have been proposed in the previous literature as drivers of mental health problems among adolescent girls [6,16,38,41,42].

From a broader perspective, our results might point to a more generalized phenomenon that will require further exploration in light of deep macrosystemic changes that have occurred in Italy in the last decade. Among these, the main two are: (1) the development of grading systems with higher educational expectations, providing the establishment of a specific evaluation referred to as the student’s conduct and the introduction of numeric marks at the elementary and middle schools; (2) the cultural transformations caused by the social media revolution [43,44,45]. Finally, Italy was severely affected by the COVID-19 pandemic, with schools being closed for over 15 months and concurrent strict social distance measures being implemented [46]. All of these elements could have precipitated and accelerated a phenomenon already visible before. Further works, including national representative data such as HBSC, could explore the impact of these measures on Italian adolescent mental health. 

To our knowledge, this is the first application of the Dual Factor Model of mental well-being to a sample of adolescents in Italy, suggesting a promising key to reading such a complex phenomenon. The present work analyzed data from a nationally representative sample of Italian adolescents recruited with a strict methodology, typical of HBSC. Moreover, the observed trend was confirmed not only by exploring the means but also at the observations level, so independently by the adopted cut-offs, thus underlining our findings’ validity. Among the limitations, there are the self-report nature and the cross-sectional nature of the data, which could partially distort the results. Furthermore, the choice of the variables has been made on the basis of the data availability of the Italian HBSC surveys and the updated literature on this topic. Moreover, a relatively narrow time period was studied to provide exhausting temporal trends because of the comparability constraints of our data. In this perspective, the work enlightens some early evidence that relevant changes in the adolescents’ mental condition could have occurred in Italy, whose implications are still largely understudied. Further investigations using a person-oriented approach are called to provide a more comprehensive meaning of dynamic aspects of adolescent mental health through the lenses of the Dual Factor Model.

## 5. Conclusions

To our knowledge, this work provides the first application of the Dual Factor Model of mental well-being to a sample of adolescents in Italy, suggesting a promising key to reading this complex phenomenon. Our findings are worthy of attention: in the last decade, Italian adolescents, especially 13–15-year-old girls, shifted from Complete Mental Health to Incomplete Mental Illness. Further research is needed to investigate this deep change in the mental well-being of adolescents in Italy and, more specifically, which elements could break up the connection between psychological symptomatology and cognitive perception of life satisfaction. We will have to understand if this dissociation could be considered a lack of recognition of the self-health problems or, in contrast, as a positive outcome of implementing good coping strategies and integration policies focused on enhancing the social integration of adolescents with psychological symptomatology.

## Figures and Tables

**Table 1:** PHC and LS across surveys, stratified by gender and age (*N* = 165,683). PHC and LS as continuous variables.

	Boys (*N* = 83,020)			Girls (*N* = 82,683)		
	11 years (*N* = 29,611) Mean (95% CI)	13 years (*N* = 28,834) Mean (95% CI)	15 years (*N* = 24,575) Mean (95% CI)	11 years (*N* = 28,427) Mean (95% CI)	13 years (*N* = 28,763) Mean (95% CI)	15 years (*N* = 25,493) Mean (95% CI)
PHC score						
Survey ¹						
2010	5.1 (4.9; 5.3)	5.2 (5.0; 5.4)	5.7 (5.5; 5.8)	5.3 (5.0; 5.5)	6.6 (6.5; 6.7))	7.5 (7.4; 7.6)
2014	5.0 (4.9; 5.1)	5.3 (5.1; 5.5)	5.8 (5.6; 5.9)	5.5 (5.3; 5.7)	7.7 (7.5; 7.8)	**8.6 (8.4; 8.7)**
2018	5.9 (5.7; 6.0)	6.0 (5.8; 6.1)	6.4 (6.3; 6.6)	6.4 (6.2; 6.6)	**8.2 (8.0; 8.4)**	**9.1 (9; 9.2)**
LS score						
Survey ¹						
2010	8.0 (7.9; 8.1)	7.7 (7.7; 7.8)	7.4 (7.3; 7.4)	8.1 (8.0; 8.2)	7.4 (7.4; 7.5)	6.9 (6.8; 7.0)
2014	7.9 (7.9; 8.0)	7.6 (7.5; 5.7)	7.2 (7.2; 7.3)	7.8 (7.7; 7.9)	7.0 (6.9; 7.1)	6.7 (6.6; 6.7)
2018	8.0 (7.9; 8.0)	7.7 (7.7; 7.8)	7.3 (7.3; 7.4)	8.0 (7.9; 8.0)	7.4 (7.3; 7.4)	6.9 (6.9; 7)

¹ Sample size in 2010, 2014 and 2018 surveys was of 58,928, 47,799, and 58,976 adolescents, respectively. Abbreviations. CI: confidence interval; LS: life satisfaction; PHC: psychological health complaints.

## Data Availability

The data presented in this study are available following the Italian HBSC data access policy. Requests should be directed to paola.nardone@iss.it, member of the National Centre for Disease Prevention and Health Promotion, Italian National Institute of Health.

## Supplementary Materials

The following supporting information can be downloaded at: https://www.mdpi.com/article/10.3390/children9121981/s1, Figure S1: Results from the sensitivity analysis on PHC score using a cut-off of 8; Figure S1: The adopted frame of the Dual Factor Model for this work.
