# Supplementary material for: The Mental Well-Being of Italian Adolescents in the Last Decade through the Lens of the Dual Factor Model
Source: Children (Basel). 2022 Dec 16;9(12):1981. doi: 10.3390/children9121981 (PMC9777362; doi:10.3390/children9121981)
Supplement: Supplementary file 1 [file children-09-01981-s001.zip › children-2000623-supplementary.pdf]

The mental well-being of Italian adolescents in the last decade through the lens of the Dual Factor Model.

Supplementary Material

Michela Bersia [1,2], Lorena Charrier[1], Paola Berchialla[3], Alina Cosma[4,5], Rosanna Irene Comoretto[1,*], and Paola Dalmasso[1].

[1] Department of Public Health and Pediatrics, University of Torino, Via Santena 5 bis, 10126 Torino, Italy
[2] Post Graduate School of Medical Statistics, University of Torino, Via Santena 5 bis, 10126 Torino, Italy
[3] Department of Clinical and Biological Sciences, University of Torino, Regione Gonzole 43, 10043 Orbassano, Italy
[4] Department of Sociology, Trinity College Dublin, 3 College Green, Ireland
[5] Sts Cyril and Methodius Faculty of Theology, Olomouc University Social Health Institute, Palacky University in Olomouc, Olomouc, Czech Republic
* Correspondence: rosannairene.comoretto@unito.it

Table of contents

Figure S1. Results from the sensitivity analysis on PHC score using a cut-off of 8……………………………………..…………………………………………………2

Figure S2. The adopted frame of the Dual Factor Model for this work……………………………………………………………..………..…………………………………..3

Figure S1. Results from the sensitivity analysis on PHC score using a cut-off of 8.

PHC2	PHC score cut-off >=8	PHC score cut-off <8	Total
>=2	54,345	4,522	58,867
<2	6,998	98,102	105,100
Total	61,343	102,624	163,967

Gold standard was defined as PHC2 = >=2 (>=2 psychological health complaints more than once a week)

Results of the sensitivity analysis

		Mean	95% Confidence Interval]	
Prevalence	Pr(A)	35.9%	35.7%	36.1%
Sensitivity	Pr(+|A)	92.3%	92.1%	92.5%
Specificity	Pr(-|N)	93.3%	93.2%	93.5%
ROC area	(Sens. + Spec.)/2	0.93	0.93	0.93
Likelihood ratio (+)	Pr(+|A)/Pr(+|N)	13.86	13.55	14.18
Likelihood ratio (-)	Pr(-|A)/Pr(-|N)	0.08	0.08	0.08
Odds ratio	LR(+)/LR(-)	168.47	162.06	175.15
Positive predictive value	Pr(A|+)	88.6%	88.3%	88.8%
Negative predictive value	Pr(N|-)	95.6%	95.5%	95.7%

Legend: PHC2: ≥2 symptoms more than once a week; PHC score cut-off: dichotomization of the continuous PHC variable adopting a cut-off value of 8.

Figure S2. The adopted frame of the Dual Factor Model for this work.

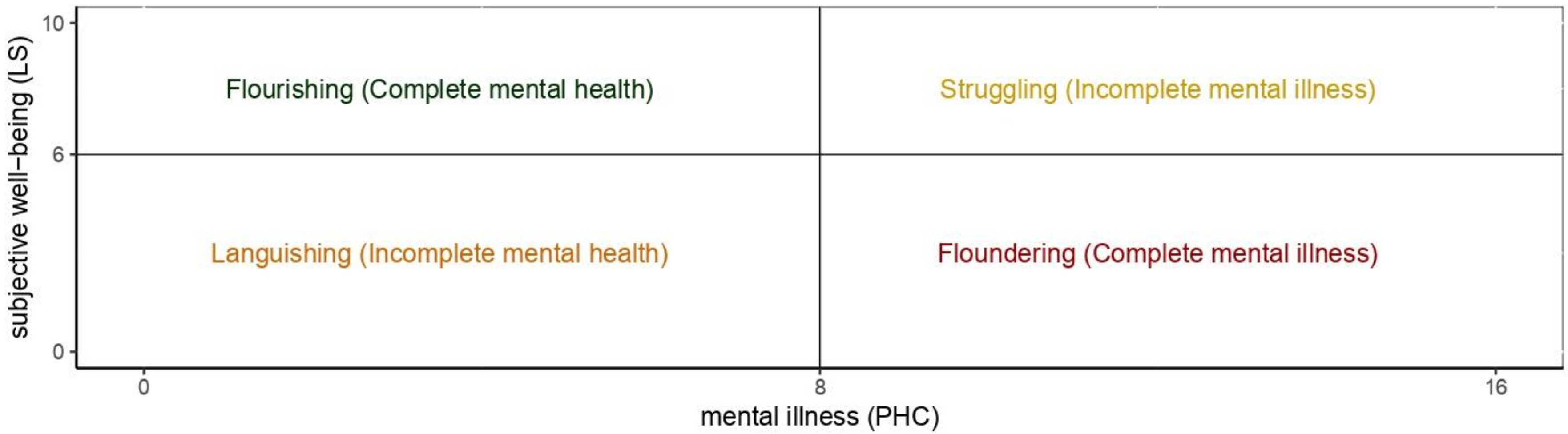

Abbreviations. LS: life satisfaction; PHC: psychological health complaints.
